# Mortality and Excess Mortality: Improving FluMOMO

**DOI:** 10.1155/2021/5582589

**Published:** 2021-06-07

**Authors:** Luís Portugal

**Affiliations:** ^1^Partner and Managing Director, Actuarial Group, Av. Duque d'Ávila, 185, 3A and 3B, 1050‐082 Lisbon, Portugal; ^2^Fellow, IAP, Campo Grande 28, 8C‐1700‐093 Lisboa, Portugal

## Abstract

FluMOMO is a universal formula to forecast mortality in 27 European countries and was developed on EuroMOMO context, http://www.euromomo.eu. The model has a trigonometric baseline and considers any upwards deviation from that to come from flu or extreme temperatures. To measure it, the model considers two variables: influenza activity and extreme temperatures. With the former, the model gives the number of deaths because of flu and with the latter the number of deaths because of extreme temperatures. In this article, we show that FluMOMO lacks important variables to be an accurate measure of all-cause mortality and flu mortality. Indeed, we found, as expected, that population ageing and exposure to the risk of death cannot be excluded from the linear predictor. We model weekly deaths as an autoregressive process (lag of one together with a lead of one week). This step allowed us to avoid FluMOMO trigonometric baseline and have a fit to weekly deaths through demographic variables. Our model uses data from Portugal between 2009 and 2020, on ISO-week basis. We use negative binomial-generalized linear models to estimate the weekly number of deaths as an alternative to traditional overdispersion Poisson. As explanatory variables were found to be statistically significant, we registered the number of deaths from the previous week, the influenza activity index, the population average age, the heat waves, the flu season, the number of deaths with COVID-19, and the population exposed to the risk of dying. Considering as excess mortality the number of deaths above the best estimate of deaths from our model, we conclude that excess mortality in 2020 (net of COVID-19 deaths, heat wave of July, and ageing) is low or inexistent. The model also allows us to have the number of deaths arising from flu and we conclude that FluMOMO is overestimating deaths from flu by 78%. Averages from the probability of dying are obtained as well as the probability of dying from flu. The latter is shown to be decreasing over time, probably due to the increase of flu vaccination. Higher mortality detected with the start of COVID-19, in March-April 2020, was probably due to COVID-19 deaths not recognized as COVID-19 deaths.

## 1. Introduction

The follow-up of mortality and excess mortality is an important tool on public health management, as it allows authorities to know how many people die, why they die, and how it trends for future years. However, mortality is not uniform during the year, and it is known that deaths increase during winter [1]. One of the key drivers that explain this is flu, especially for people aged 65 or more [2]. Years with more frequency or severity on flu have more deaths, but countries lack statistics that allow them to know exactly the mortality caused by flu. Because of that, mathematical formulas are used to estimate the number of deaths that come from flu and to measure the expected mortality and excess mortality [3].

In Europe, EuroMOMO, the network for European monitoring of excess mortality for public health action (http://www.euromomo.eu) is following weekly all-cause excess mortality in 27 European countries. For that, they use FluMOMO, which allows death estimation and excess mortality analysis. The work started with the 2009/2010 flu using the Serfling method [4]. This method developed a framework to have a standard curve of “expected seasonal mortality” and to measure the excess of deaths in respect to that baseline. The method is a mathematical formula using trigonometry to have a cyclical regression. There are no indications for mortality risk factors, such as demographic, meteorological, and health explanatory variables. Recently, the methodology was updated, and several changes were done [5]. Deaths are now considered in the context of generalized linear models with Poisson overdispersion and two explanatory variables are added to the model (extreme temperatures and the influenza activity index). The result is a change on baseline levels, as the latter starts including (as an addition) expected deaths coming from the increase of influenza activity and extreme temperatures (in summer or winter). This framework is now being used to decompose the total number of deaths into those attributable to one or more infectious pathogens circulating in a population (as COVID-19) and those attributable to deaths due to excess temperatures and other seasonal patterns [6]. We do not approach this decomposition in this paper.

This model has some problems when applied, directly, to Portugal (and probably to other countries). First, our population is getting old, and between 2009 and 2019, the population average age increased from 40 to 45 years [7]. Because of this, the average mortality rate is increasing every year. This means that from the deaths we register every year, part of it is the natural evolution of mortality because of age effect and is not related to baselines, temperatures, and flu activity.

Second, the population is not completely stable, and the number of deaths depends on the exposure of this population to the risk of dying. If the population decreases/increases, there may be fewer/more deaths in that year. Once again, it is not related to baselines, influenza activity, and extreme temperatures.

The purpose of this paper is to present one model that overcomes these problems and to give a contribution to FluMOMO improvement using demographic and health variables.

## 2. Methods

### 2.1. Data Sources

Demographic data on population and its average age were obtained from the Portuguese National Institute of Statistics [7]. Exposure to risk is the population at the beginning of the week. Population weekly mortality data, from all causes, relates to online daily mortality, provided by Health Ministry [8]. The mortality rate is the number of deaths divided by exposure to risk.

Influenza activity index is published by Portuguese Instituto Nacional de Saúde (INSA) weekly bulletins from 2009 to 2020 [9]. The index summarizes the number of influenza cases detected on the national health system. It is an important indicator because it has some statistical peaks that completely match with peaks on mortality and it is well known that influenza is an important cause of deaths during the winter [2].

Between 2009 and 2020, two heat waves are considered: the one from 2013, as it is documented on INSA (2013), and the one from July 2020, not yet documented but visible on mortality data and registered temperatures ([8] affected mortality on ISO-weeks 27 till 30).

Deaths due to COVID-19 were obtained from 2020 daily bulletins on COVID-19 deaths [10]. We need this variable because COVID-19 brought some extra mortality in 2020 and the model most accommodates for that [6]. At the same time, this gives an indication if COVID-19 pressure on hospitals generated more mortality on non-COVID-19 patients.

Flu season was considered to include ISO-weeks from week 40 of year *x* to week 20 of year *x *+* *1.

### 2.2. Methodology

FluMOMO model is given by the following equation [5]:

(1) $$\begin{array}{c} \log\left(D_{t}\right)=b_{1}\times \text{baseline}+\underset{\text{dIA}}{\sum }\left(\underset{s}{\sum }b_{2\;dIA,s}\times IA_{t-dIA,s}\right)+\underset{\text{dET}}{\sum }\left(\underset{p}{\sum }b_{3\text{dET},p}\times {\text{ET}}_{t-\text{dET},p}\right)+\varepsilon_{t}. \end{array}$$

The model uses a log link to explain deaths per week *t*, *D*_*t*_. Length of the lags is predefined as external parameters, and dIA and dET represent the delay in the formula. IA is the influenza activity index and ET represents extreme temperatures. The baseline consists of a trend and seasonality expressed as two sine waves of one-year and half-year periods. *ε*_*t*_ is the error in week *t*. See [5] for more details.

In our model, we also consider as the dependent variable (response variable) deaths per week *t*, *D*_*t*_. However, the latter will be a function of several predictors: previous week deaths, *D*_*t*−1_, deaths from the following week, *D*_*t*+1_, week influenza activity index, IA_*t*_, COVID-19 weekly deaths, *CoV*_*t*_, population average age on that week, AA_*t*_, and two factors: existence or not of week flu season FS_*t*_, and existence or not of heat wave, HW_*t*_. There is also an intercept *a*_0_ (which includes cases without heat wave and outside flu season) and an offset, the log from exposure to risk, the population number on that week, *P*_*t*_. The model also includes a weekly error *ε*_*t*_, and a log link is considered.

Several parameters need to be estimated using generalized linear models (GLMs). For an introduction to the GLM, see McCullagh and Nelder [11]. The following equation summarizes our model:

(2) $$\begin{array}{c} \log\left(D_{t}\right)=\log\left(P_{t}\right)+a_{0}+a_{1}\times D_{t-1}+a_{2}\times D_{t+1}+a_{3}\times IA_{t}+a_{4}\times CoV_{t}+a_{5}\times {\text{AA}}_{t}+a_{6}\times {\text{FS}}_{t}+a_{7}\times {\text{HW}}_{t}+\varepsilon_{t}, \end{array}$$

(3) $$\begin{array}{c} E\left(\varepsilon_{t}\right)=0,E\left(\varepsilon_{t},\varepsilon_{s}\right)=\text{constant}. \end{array}$$

Previous weeks' deaths (*D*_*t*−1_) allow us to avoid the use of trigonometric variables and deaths lead of one week (*D*_*t*−1_) is related to nowcasting from authorities to correct delays on death notifications. If they underestimate/overestimate deaths in one week, there may be an overestimation/underestimation in the next week because most of the notifications are done within a week. This is only known in the next week and one possible way to catch this effect is with this lead. Population average age introduces the age effect, and population considers the exposure to risk effect. We introduce several variables that catch the health and meteorological effects: the index of influenza activity and two factors, the existence or not of high temperatures (heat waves) and the season classification (between week 40 from one year and week 20 on the following year vs. other weeks). The number of COVID-19 deaths per week was also included, as this variable produced an addition on the 2020 weekly deaths from all causes.

We use weekly data, classified as ISO-weeks from 2009 to 2020. We estimated several regressions using Poisson distribution, overdispersion Poisson, and negative binomial distribution. The latter was chosen because of the existence of overdispersion on mortality weekly data, mainly due to the omission of some explanatory variables not completely known and the possibility of having Akaike Information Criteria (AIC) statistic for model comparison. Explanatory variables considered are analysed using z-scores and expected signals. Models within the negative binomial distribution (with different lags and explanatory variables) are selected using AIC.

Baseline is the expected value of the number of deaths obtained from the generalized linear model (GLM) considered, the negative binomial distribution, instead of overdispersion Poisson. The former also includes overdispersion and has the advantage of being more complete, as we need to estimate one more parameter, and it has several statistics available to compare different models. We consider as excess mortality all deaths above the expected deaths best estimates coming from our model. This is the same approach as FluMOMO; however, their model is different, as explained above.

## 3. Results

In Table 1, results obtained are presented. R software 4.0.3 version was used.

We conclude from Table 1 (see *Z* value) that all variables (except for COVID-19 deaths) are statistically significant and with signs according to expectations: increasing deaths on the previous week, influenza activity index, average age, flu season, and heat wave, increasing the number of deaths on that week. *P* values are very low, which shows that, to refuse these variables, the confidence level should be almost 100%. AIC was 7671.8, the lowest obtained on all model variants considered. The variants considered included different lags for deaths of previous weeks, influenza activity index and average age, and exclusion of some variables. COVID-19 deaths are not significant because they just exist in one year, 2020, and their effect may already be captured by deaths lead. This can be seen in the model without this lead in Table 2.

This model has a good explanatory power, but when we do the fit, it presents, in some weeks, some delay to match observed deaths. As explained before, this may be a consequence of deaths nowcasting and is overcome with the introduction of the lead, as can be seen in equation (2).

**Figure 1:**
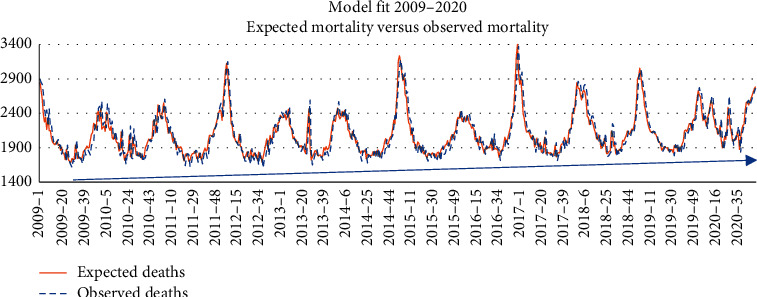
Model fit between 2009 and 2020.

In Figure 1, we may see the fit between Table 1 model deaths prediction and the observed deaths between 2009 and 2020 on ISO-week basis. The fit is very good and shows the increasing trend of mortality produced by ageing.

**Figure 2:**
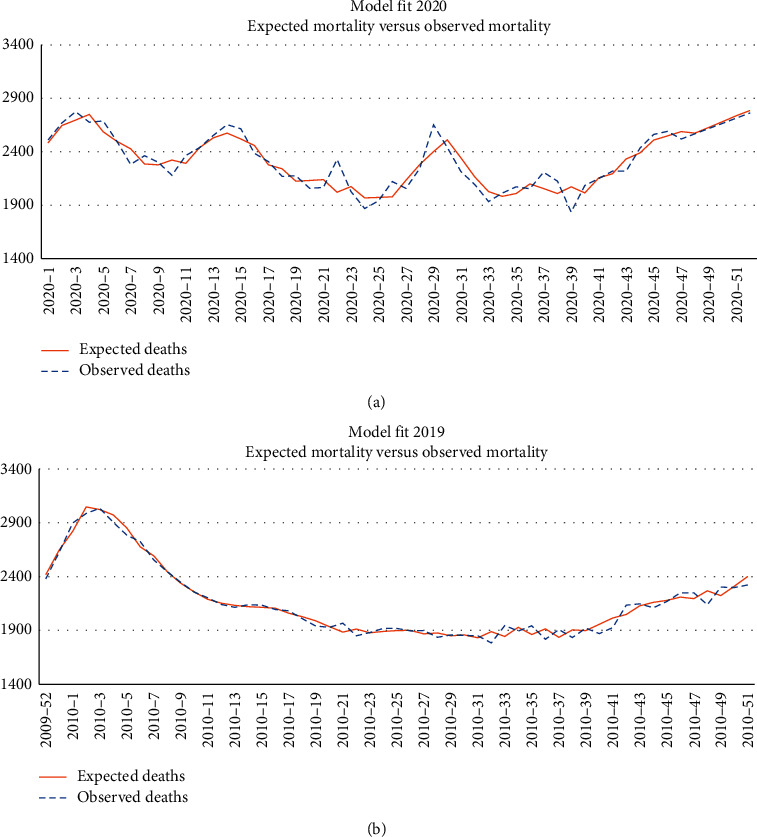
Model fit for 2019 and 2020.

Figure 2 shows the same fit just for 2019 and 2020. We can see that the fit is very good. However, some differences arise between 2019 and 2020. In the latter, there is one pandemic, COVID-19, and a heat wave in July.

In respect of April mortality, one study [12] identified from 2400 to 4000 deaths of excess mortality due to the lockdown effect (people were afraid of going to hospitals). However, the study baseline was August mortality and not April mortality. Also, had the lockdown been the cause of these deaths the same should have happened in the following months (which was not the case). The fit in Figure 2 does not show regular excess mortality.

### 3.1. Excess Mortality

**Figure 3:**
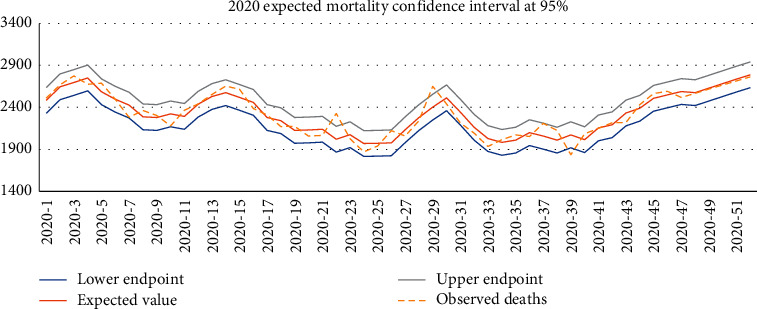
Expected deaths 95% confidence interval for 2020.

For 2020, we realize that the mortality observed is mostly inside the confidence interval of expected mortality. This means that the expected mortality (which also includes COVID-19 deaths and one heat wave in July) is inside the 95% confidence interval of expected values (Figure 3).

Of course, the conclusions could change if we considered a narrow confidence level with a lower error.

Analysing 2020 mortality, we concluded that we had 123,578 deaths during the year, compared with 112,373 in 2019 and 113,597 in 2018. Apparently, we had important excess mortality in 2020, when compared with the last two years, between 9,981 and 11,205 deaths. However, this comparison is not correct. We must start by deducting in 2020 COVID-19 deaths, 6906, because this disease was not active in 2018 and 2019. When we do that, the gap narrows. Now, it is between 1,384 and 2,608 deaths and we must be aware that we had a heat wave in 2020 and some ageing effect during the year.

Using our second model, as in Table 2, we may calculate the impact of these three variables on 2020 deaths and then we will be able to compare it with 2018 and 2019. This means, in Table 3, an excess mortality, that is unexpected mortality, between −540 and +684 deaths, depending on the year is considered as a benchmark (2018 or 2019).

This is almost zero and much lower than the figures spread on the media (around 6000 deaths of excess mortality). From both figures, we think it is wise to say that there was not (net of the mentioned effects) excess mortality and if there was, it was not so high. This is in line with other models fitted where the COVID-19 coefficient was highly significant and with the exponential of its parameter equal to one.

### 3.2. Deaths from Flu

FluMOMO has a big advantage as a model: it allows, directly, to estimate the number of deaths allocated to flu. These deaths are the ones arising above the baseline from weeks with mortality higher than baseline (that are not a consequence of extreme temperatures). The problem is that any model error, parameter error, and random error from FluMOMO will be considered deaths from flu if there is excess mortality above the baseline (not considered as coming from extreme temperatures).

Our model allows us to know deaths that should be considered as caused by flu and will reduce model error and parameter error. They are reduced because the model considers extra variables which are important on mortality explanation. To have an estimate for deaths that arise from flu, we just need to run our model considering Influenza Activity as zero every week (using coefficients of Table 1).

In Table 4, we may see our forecast of deaths due to flu, 1872 in 2018/2919 and 1,247 in 2019/2020. The first figure compares with the FluMOMO official estimate of 3,331 deaths (public forecasts for 2019/2020 are not yet available).

We identified the following reasons for such a difference:

- FluMOMO considers as deaths from flu all deaths above baseline if extreme temperatures did not occur on that day. The baseline is just an historical fit with cosines and sines without any demographic, health, or meteorological meaning. Our model considers explanatory variables to explain deaths and does not have a mathematical baseline.
- FluMOMO uses model deviations to arrive to deaths by flu through the influenza activity index. Our model connects deaths by flu to influenza activity index after identifying its contribution inside all other variables considered in the model (see Table 1).
- Several important variables are not used by FluMOMO, as population ageing, the level of exposure to the risk of dying, and the deaths from previous week.

### 3.3. Average Probability of Death

**Figure 4:**
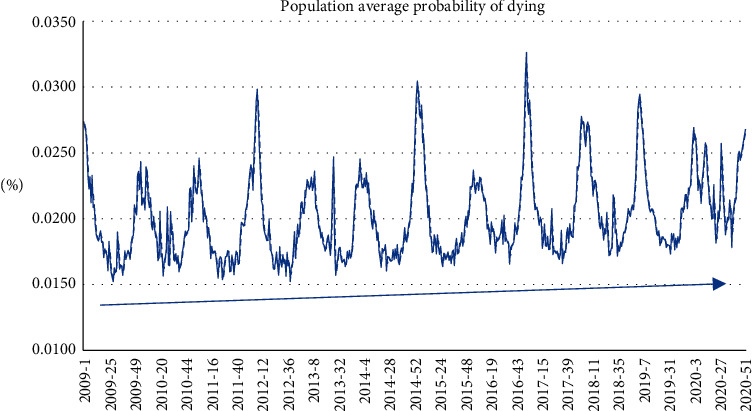
Average weekly probability of dying.

The model also allows us to have the population's average weekly probability of dying (Figure 4).

**Figure 5:**
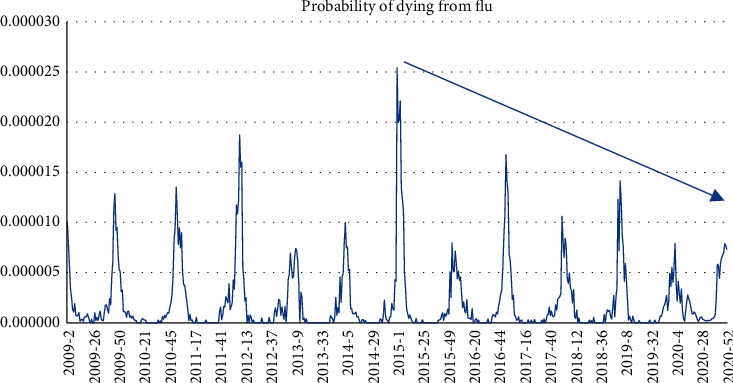
Average probability of dying from flu.

The same can be done to the probability of dying by flu (Figure 5). As we can see, there is a decrease of such probability over time, probably due to the increase in flu vaccinations.

## 4. Conclusions

The model presented shows that there is scope to FluMOMO improvement. Using negative binomial regression instead of overdispersion Poisson is not the key issue but allows model comparisons using AIC (which is important when we have several explanatory variables). However, changing the mathematical baseline to a more demographic, meteorological, and health-based approach to mortality allows us to better understand mortality and to forecast deaths and deaths allocated to flu with more accuracy.

Excess mortality analysis will be straightforward and easier to explain with this model. This model has the same degree of difficulty to apply than traditional FluMOMO. The model also shows that not only are deaths by flu lesser than those presented in traditional FluMOMO (which overestimate them as 78%), but the probability of dying by flu is decreasing over time.

Finally, the model also helps to understand deviations from expected mortality.

## 5. Discussion

It is not possible to compare this study with other studies because FluMOMO was just updated in 2018 and there are not yet published works in this respect. However, comparison with FluMOMO, within our framework, did not show evidence of lag effects considered by FluMOMO for influenza activity. It was not possible to do the same analysis for extreme temperatures due to the absence of common data for that.

The main limitation of this work is that we do not identify different models for different age bands. We are aware that age effect, influenza activity index, heat wave effects, and COVID-19 deaths also depend on age bands. Indeed, several improvements could be done with this model.

Firstly, to consider age bands, our model is estimated to all the age bands together. This could show different behaviours of this model, as the age effect is not the same for all age bands. Secondly, we did not split the study between genders. Age effects may be different when we consider it. Finally, other variables could be used, as the minimum and maximum temperatures. This could help to understand influenza activity index and heat waves.

The autoregression considered shows some accuracy. Eventually, other nonlinear structures could be considered, but the attempts we did to improve the results, with different lag structures, were not successful.

Age distribution in Portugal was considered to have a linear impact on deaths. This was done because the evolution, since 2009, had been linear. For the period between 2009 and 2020, it was easy to do the fit because the probability of dying in Portugal increased linearly.

However, it is possible that death's relation with ageing becomes nonlinear as the population average age increases above some threshold.

The effect of influenza on mortality may vary from season to season and we did not consider that completely because we just used two seasons (flu season and no-flu season). The former is an objective variable, as flu season is always defined as ISO-weeks from week 40 of year *x* to week 20 of year *x* +  1. To compensate any deviation, heat waves were introduced. The latter may be subjective, but it is very important to understand mortality as it was the case in July 2020.

## Figures and Tables

**Table 1:** Negative binomial fit log link and offset on exposure to risk with lead.

Generalized linear model to explain the number of deaths per week				
Explanatory variables	Coefficients	Standard error	*Z* value	*P* value
Intercept	−9.82399	0.05193	−189.183	2.00*E* − 16
Deaths from previous week	0.00022	0.00001	16.222	2.00*E* – 16
Deaths from the following week	0.00020	0.00001	20.910	2.00*E* − 16
Influenza activity index	0.00019	0.00010	1.896	6.16*E* − 02
COVID-19 deaths	0.00001	0.00003	0.295	7.68*E* − 01
Average age	0.00973	0.00130	7.497	2.00*E* − 14
Existence of flu season	0.02181	0.00467	4.673	2.97*E* − 06
Existence of heat wave	0.05380	0.01741	3.091	2.00*E* − 03

**Table 2:** Negative binomial fit log link and offset on exposure to risk without lead.

Generalized linear model to explain the number of deaths per week				
Explanatory variables	Coefficients	Standard error	*Z* value	*P* value
Intercept	−9.874893	0.061893	−159.548	2.00*E* − 16
Deaths of previous week	0.000319	0.000010	32.138	2.00*E* − 16
Influenza activity index	0.000936	0.000106	8.844	2.00*E* − 16
COVID-19 deaths	0.000107	0.000037	2.936	3.32*E* − 03
Average age	0.014851	0.001502	9.885	2.00*E* − 16
Existence of flu season	0.041480	0.005363	7.734	1.04*E* − 14
Existence of heat wave	0.117854	0.117854	5.817	6.00*E* − 09

**Table 3:** Low or inexistence of excess mortality in 2020.

Excess mortality in 2020		
(A)	Deaths 2020	**123 578**
(B)	COVID-19 deaths	6 906
(C)	Ageing effect deaths	2 401
(D)	July heat wave deaths	1 213
**(E) = (A) **−** (B) **−** (C) **−** (D)**	**Net deaths 2020**	**113 057**
(F)	Deaths 2019	112 373
(G)	Deaths 2018	113 597

Excess mortality		

(E)** **−** **(F)	In respect to 2019	684
(E)** **−** **(G)	In respect to 2018	−540

**Table 4:** Estimated deaths by flu from 2018 to 40 until 2019-20.

Deaths in Portugal	Flu season from week 40 to week 20	
	2018/2019	2019/2020
With influenza activity on model	70 226	70 386
Assuming no influenza activity	68 354	69 139
**Our model estimated deaths by flu**	**1 872**	**1 247**
**FluMOMO estimated deaths by flu**	3 331	na

## Data Availability

Data are available upon request to the corresponding author.
